# Differences in microRNA expression between melanoma and healthy adjacent skin

**DOI:** 10.1186/s12895-018-0081-1

**Published:** 2019-01-05

**Authors:** Mariya Aksenenko, Nadezhda Palkina, Anna Komina, Liubov Tashireva, Tatiana Ruksha

**Affiliations:** 1 Department of Pathophysiology, Krasnoyarsk State Medical University, Krasnoyarsk, 660022 Russia; 2 Department of Pathological Anatomy and Cytology, Cancer Research Institute, Tomsk National Research Medical Center of the Russian Academy of Sciences, Tomsk, 634009 Russia

## Abstract

**Background:**

The tumor microenvironment is composed of cancer-associated fibroblasts, tumor-associated macrophages, endothelial cells, immune cells, signaling molecules and extracellular matrix structures, which closelycommunicate with the tumor via multiple mechanisms. MicroRNAs are paracrine regulators that provide a direct interaction between the microenvironment and cancer cells. In the presentstudy, we aimed to identify the microRNA expression profile in melanoma compared with thatin healthy adjacent skin, with a further assessment of altered microRNA signaling pathways and target genes.

**Methods:**

Formalin-fixed paraffin-embedded (FFPE) melanoma tissue samples were separated by dissection into tumor and surrounding health tissue fragments. MicroRNA expression profiles were obtained by microarray using Gene Atlas Microarray System (Affymetrix, California, USA). To confirm microarray results real-time PCR was carried out. Bioinformatic analysis was performed using the DIANA-miRPath v.3.0 database. Target genes for miR-146a-5p were determined using three algorithms: TargetScan 7.0, miRWalk 2.0 and miRTarBase v.4.5.

**Results:**

A microarray profiling revealed 143 microRNAs asdifferent in tumor versus adjacent tissues. Expression level of hsa-miR-146a-5p showedto be higher in melanoma cells as compared to thehealthy adjacent skin. The bioinformatic study has determined several signaling cascades associated with miR-146a-5p:Toll-like receptor pathway, NF-κB pathway, ErB pathway, and measles signaling pathway. The 38 target genes have been shown for miR-146a-5p of which NRAS gene is known asone of the most frequent mutated in melanoma.

**Conclusions:**

Elucidation of the role of miR-146-a-5p in complex interactions between the tumor and the cells of healthy adjacent skin is necessary for our understanding of the mechanisms oftumor progression. Significant differences found between cancer cells and adjacent tissues in microRNA expression profile corresponding to divergent mRNA/protein levels in these structures should be taken into account when tumor samples characterization estimatedby high-throughput methods.

**Electronic supplementary material:**

The online version of this article (10.1186/s12895-018-0081-1) contains supplementary material, which is available to authorized users.

## Background

The tumor microenvironment consists of soluble factors, extracellular matrix and non-tumor cells,such as cancer-associated fibroblasts, tumor-associated macrophages, pericytes, endothelial cells and infiltrating immune cells [1]. The tumor microenvironment affects tumor cells through triggering various signaling cascades, by releasingintercellularadhesion molecules and cytokines [2]. Tumor cells recruit stromal cells by secreting chemokines and growth factors, which educate them into creating a tumor-favoring community [3]. These effects are observed universally and are not dependenton the type of tumor [4]. Advances in cancer research have highlighted the significance of the dynamic evolution of the tumor microenvironment, whichaffectstumor formation, dissemination and resistance totreatment.

The ability of melanoma cells to interact with microenvironment components contributes to a shift in the biological behavior of the tumor; in particular, it helps reduce the tumor cell response to apoptotic stimuli [5]. Tumor microenvironment status is dependent on various factors, including oxidative stress, pH dynamics and acidosis (a decrease in the number of metabolites entering the cells). Collectively, all these factors contribute to the developmentof genome instability in melanoma cells [6]. It is also known that the changes in the tumor microenvironment are inhomogeneous, which is due to the heterogeneous origin of tumor cells and their dynamical behavior [7]. Recent studies proposed that disrupting the suppressive effects of the tumor microenvironment allows melanoma to progress from locally confinedto invasive [8].

One mechanism through which cancer cells promote disease progression is alteration oftheir miRNA expression profile. MicroRNAs are small non-coding RNAs playing a key rolein post-transcriptional gene regulation in diverse biological processes [9]. MicroRNAs regulate gene expression by binding to the 3′-untranslated regionof target mRNAs, thusresultingin translational repression or RNA degradation [10]. One microRNA may simultaneously regulate a set of genes, thereby controllingmultiple signaling pathways [11]. These molecules may act as tumor suppressors or oncogenes. To achieve this, microRNAs produced by tumor microenvironment cells may act as paracrine modulators of thebiological behavior of cancer cells; they regulate gene expression at the post-transcriptional level, thereby controlling cellular processes such as differentiation, proliferation and migration [12]. In addition, microRNAs have emerged as important contributors to the balance of pro- and anti-angiogenic factors [13]. The expression patterns of microRNAs are tissue-specific [14], suggesting their potential valueas clinical biomarkers [15].

The aim of the present study was to identify the differences between the microRNA expression profile in melanoma and healthy adjacent skin, and to further assess the altered microRNA signaling pathways and target genes.

## Methods

### Tissue samples

The study protocol was approved by the Local Ethics Committee of Krasnoyarsk State Medical University (protocol no. 70/2016, issued on June 7, 2016). All procedures performed in studies involving human participants were in accordance with the ethical standards of the institutional and/or national research committee and with the principles outlined in the 1964 Declaration of Helsinki and its later amendments or comparable ethical standards. Informed written consent was obtained from all individual participants included in the study. A total of 16formalin-fixed, paraffin-embedded (FFPE) malignant melanoma tissue samples from Caucasian patients were investigated. The tumors were obtained from the Krasnoyarsk Regional Pathologic Anatomy Bureau, where diagnosis was established by a certified pathologist. The histopathological criteria for melanoma diagnosis referred to the presence of melanocytes in the skin, their cytological characteristics, architectural and organizational disposition. Melanoma patients were aged 36–81 years; 43.75% of the patients were female and 56.25% weremale. Superficial spreading melanoma was diagnosedin 18.75% of the patients (*n* = 3), nodular melanoma in 18.75% (*n* = 3), lentigo maligna melanoma in18.75% (*n* = 3), acral lentiginous melanoma in 25.00% (*n* = 4), and the type of tumor was undetermined in 18.75% (*n* = 3) ofthe cases. Melanoma of the trunk (skin of the back, chest and abdomen)occurred in 37.50% (*n* = 6) of the patients, 6.25% (*n* = 1) had tumorsof the head and neck, and 56.25% (*n* = 9)of melanomas were diagnosed in the extremities. Skin samples obtained from the back of healthy volunteers (*n* = 5) were used as the control group.

The 3–4-μm thick sections were stained with hematoxylin and eosin and examined by light microscopy. A morphological evaluation was then performedtodeterminethe feasibilityof separating the tumor and the surrounding non-cancerous tissues. The melanoma samples were macrodissected at room temperature on 5 × 10-*μ*m slidesby removing healthy adjacent tissues. For this purpose, the area of interest was marked and scratched off using a sterile, single-use scalpel correspondingly to hematoxylin and eosin-stained template slide.

### Laser microdissection

FFPE melanoma samples were prepared by a microtome MR2255 (Leica,Norcross, GA, USA) into7-μm slides (Biovitrum, Moscow, Russia) and dried in Thermostat TS-1/20 (SPU, Smolensk, Russia) for 12 h. To remove the paraffin, the slides were placed in xylene (at a temperature of 39 °C) 3 times for 10 min each time, in alcohol (96 °C) 3 times for 5 min each time, and then in distilled water once for 10 min. Then, the rehydrated sectionswerestained with hematoxylin (Biovitrum) and air-dried. Isolation of tumor cells and adjacent non-cancerous tissue cells was performed on a PALM Microbeam laser microdissector (CarlZeiss, Jena, Germany). Tumor cells were identified as irregular melanocytes with cytological atypia, accompanied by aberrant architectural appearance, and some melanocytes exhibited nuclear atypia. Non-cancerous cells were identifiedout of the tumor nests and morphologically corresponded to fibroblasts, histiocytes and lymphocytes. At least 10,000 tumor cells and 10,000 cells ofhealthy adjacent skin from each slide were separately cut and placed in tubes containing the RNA stabilizer RNAlater® Solution (Thermo Fisher Scientific Inc., Waltham, MA, USA). The isolated cells were stored at − 20 °C.

### RNA isolation

Total RNA was obtained by the Recover All™ Total Nucleic Acid Isolation kit (Ambion, Life Technologies, Vilnius, Lithuania). FFPE tumor samples were deparaffinized with xylene and rehydrated with 96% ethanol. Then, the samples were incubated in digestion buffer. The microRNA concentration was quantified on Qubit 2.0 fluorimeter (Invitrogen by Life Technologies; Thermo Fisher Scientific Inc., Singapore) by the use the Qubit™ microRNA Assay kit (Ref. Q32880, Invitrogen; Thermo Fisher Scientific Inc., Eugene, OR, USA). In accordance with themanufacture’s recommendationsto use 100 ng as the minimal input amount of microRNA, the concentration of microRNA in the samples submitted to microarray analysis exceeded 16.3 ng/μl.

### Microarray

The microarray analysis was performed on the GeneAtlas™ Microarray System (Affymetrix). Total RNA from each samplewas labeled with biotin in accordance to Affymetrix Flash Tag™ Biotin HSR (Ref. 901,913, Affymetrix) kit instructions. The labeled molecules were hybridized onto Affymetrix miRNA 4.1 Array Strip (Affymetrix) using the GeneChip GeneAtlas™ Hybridization and Stain Module (Ref. 902,135) reagents (Affymetrix). After hybridization and washing, the arrays were scored on the Imaging Station of the GeneAtlas™ Microarray System. Array data of the samples have been deposited in the Array Express database at EMBL-EBI (www.ebi.ac.uk/arraexpress) under the accession no. E-MTAB-7060.

### TaqMan miRNA quantitative PCR

To confirm the microarray results, total RNA previously isolated from melanoma and healthy adjacent skin samples wasused to estimate the expression levels of miR-363-3p and miR-3591. These microRNAs were selectedasmiR-363-3p was upregulated and miR-3591 wasnot altered according to the microarray results. Then, for a more detailed investigation of miR-363-3p, miR-18a-5p and miR-146a-5p in melanoma and surrounding tissues,the material obtained by microdissection was used. The aforementioned microRNAs were selected because their expression in melanoma was altered by > 4-fold compared with that inhealthy adjacentskin:miR-363-3p (fold change, 5.43), miR-18a (fold change, 4.01) and miR-146a-5p (fold change, 8.94). The target genes of these miRNAs have been associated with carcinogenesis: Cell cycle-regulating genes (including signaling cascade MAPK genes) and cancer pathway genes (including genes regulating cell proliferation, differentiation and angiogenesis). RNA was isolated by Ribo-Zol B kit (AmpliSens, Moscow, Russia) in accordance withthe manufacturer’s recommendations and reverse transcribed using 0.1 μl of 5x primers miRNA TaqMan assays specific to the investigated microRNA and endogenous controls (Cat. nos. 4427975 and001973, Applied Biosystems, Foster City, CA, USA), 0.4 μl RT-buffer, 0.4 *μ*l dNTPs, 0.15 *μ*l nuclease-free water and 0.05 *μ*l revertase enzyme from the Reverta kit (AmpliSens) per 1 *μ*l of isolated RNA. The reaction mixture was incubated at 37 °C for 30 min. The cDNA was proceeded for the qPCR or stored at − 20 °C. PCR was performed with 8 μl of the 2.5-fold qPCR reaction mixture and ROX reference dye (Syntol, Moscow, Russia), 1 *μ*l of 20x primers for miR-363-3p, miR-3591, miR-18a-5p, or miR-146a-5pfrom TaqMan™ MicroRNA Assay (Cat. no. 4427975, Applied Biosystems) and nuclease-free water, to a total volume of 20 *μ*l. U6snRNA and RNU6B (Cat.no. 4427975, Assay IDs 001973 and 001093, respectively;Applied Biosystems) served as endogenous controls. Thermalcycling conditions were as follows: 50 °C for 2 min and 95 °C for 10 min, 40 cycles at 95°C for 15 s and at 60 °C for 1 min with FAM detection. All real-time PCRs were carried out in a StepOne™ Real-Time PCR System (Applied Biosystems, Singapore) in triplicate. Relative microRNA expression levels were determinedusing ΔCtmethod after normalization with the geometric mean of U6snRNA and RNU6B.

### Kyoto encyclopedia of genes and genomes (KEGG) pathway analysis of miRNA target genes

KEGG pathway analysis based on microRNA signature was performed with DIANA-mirPath v.3.0. MicroRNA targets were predicted with the use DIANA-microT-CDS computational tool. The threshold (*P* ≤ 0.05) and false discovery rate (FDR) (*P* ≤ 0.05) were determined using Fisher’s exact test. Three different algorithms, namely TargetScan 7.0, miRWalk 2.0 and miRTarBase v.4.5, were applied to identify the validated targets of miR-146a-5p. Genes mentioned by all three aforementioned tools were validated as microRNAs targets to analyze further. Then the genes were sorted according to the Cumulative Context Score. The PANTHER™ v.10.0 classification system was applied to interpret the biological function of the validated targets of miR 146a-5p. The criteria for selecting miR-146a-5p were as follows: the presence of common genes for the three abovementioned databases and the presence of target genes participating in carcinogenesis among them.

### Statistical analysis

The microarray data were processed with Expression Console and Transcriptome Analysis Console 3.0 software by Affymetrix. Statistical analysis was done using ANOVA test and FDR-corrected values. qPCR data were analyzed by the ΔCt method. Mann-Whitney *U*-test with the Statistica 6.1 software (Stat Soft, Moscow, Russia) was applied to evaluate significant differences between two groups. *P* < 0.05 was determined as statistically significant.

## Results

### MicroRNA expression in tumor and microenvironment from FFPE tissues

**Fig. 1:**
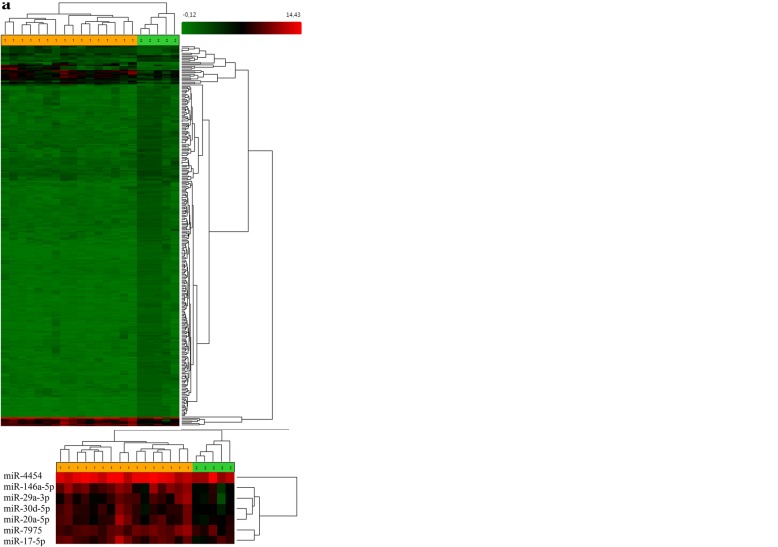
(**a**) Unsupervised hierarchical clustering analysis of microRNAs differentially expressed between melanoma tissues andhealthyadjacent skin. Heatmap colors reflect the expression levels of microRNAs according to a color key (from green to red). (1) Melanoma samples (marked in yellow); (2) surrounding adjacent tissue (marked in green) (**b**) Unsupervised hierarchical clustering analysis of microRNAs differentially expressed in melanoma, healthy adjacent tissues and normal skin. Heatmap colors reflect the expression levels of microRNAs according to a color key (from green to red). (1) Melanoma samples (marked in green); (2) surrounding adjacent tissue (marked in yellow) and normal skin (marked in orange).

A microarray analysis identified that the expression of 143 microRNAs was altered in melanoma tumor cells compared with that in healthy adjacent skin (Fig. 1a) following FDR-correction (Additional file 1). A total of 32 microRNAs were upregulated and 111 were downregulated in melanoma. A microarray analysis identified 1140 microRNAs that were altered in the three groups studied: healthy skin of control volunteers, melanoma tissues and healthy adjacent skin. Hierarchical clustering revealed evident separation into clusters corresponding to the three groups studied (Fig. 1b). Following hierarchical clustering, we observed a separate micro-cluster appearing as a red cluster at the bottom of the diagram, where the highest level of microRNA expression was marked; this micro-cluster corresponded to 5 microRNAs that were differentially expressed in the three groups, and was composed of miR-146a-5p (FDR = 0.000003), miR-4454 (FDR = 0.00001), miR-29a-3p (FDR = 0.000158), miR-30d-5p (FDR = 0.000081), miR-20a-5p (FDR = 0.000539), miR-7975 (FDR = 9.33E-07) and miR-17-5p (FDR = 0.000251).

Hierarchical clustering of melanoma and healthy adjacent skin profiles revealed a separate cluster that corresponded to 7 microRNAs, the expression of which was higher in melanoma compared with thatin the adjacent skin. The cluster was composed of miR-146a-5p (fold change, 8.94; FDR = 0.03), miR-4454 (fold change, 3.76; FDR = 0.035), miR-7975 (fold change, 2.64; FDR = 0.035), miR-17-5p (fold change, 2.95; FDR = 0.035), miR-29a-3p (fold change, 2.95; FDR = 0.02), miR-30d-5p (fold change, 2.24; FDR = 0.03) and miR-20a-5p (fold change, 2.05; FDR = 0.04) (Additional file 2). Bioinformatic analysis revealed that microRNAs related to this cluster were involved in the regulation of signaling pathways implicated in carcinogenesis: TGF-β signaling pathway, p-53 signaling pathway, extracellular matrix receptor interaction, proteoglycans in cancer andcell cycle signaling pathway (Additional file 3).

The highest differences in microRNA expression levels between melanoma and healthy adjacent tissues were found in the upregulated miR-138-5p, miR-146b-5p, miR-664b-3p, miR-146a-5p and miR-509-3-5p, and the downregulated miR-877-3p, miR-4300, miR-4720-3p and miR-6761-5p. Bioinformatics analysis of microRNAs that differed the most among the three study groups identified their involvement in the regulation of 28 signaling pathways. Most of the signaling pathways were implicated in carcinogenesis (Table 1).

**Table 1:** Signaling pathways under control of differently expressed microRNAs in melanoma, healthy adjacent skin, and normal skin

1 №	2 KEGG pathway/Pathway ID	3 *P*-value	4 #Genes	5 #miRNAs
1.	Fatty acid metabolism (hsa 01212)	1.9665081572e-20	16	2
2.	Fatty acid biosynthesis (hsa 00061)	3.88343729046e-12	4	1
3.	Fatty acid degradation (hsa 00071)	1.1705763613e-07	13	2
4.	Viral carcinogenesis (hsa 05203)	8.9531652961e-07	58	3
5.	Cell cycle (hsa04110)	1.46761453192e-05	44	3
6.	Thyroid hormone signaling pathway (hsa 04919)	0.00023866494053	34	3
7.	Lysine degradation (hsa 00310)	0.000277746132555	18	2
8.	Fatty acid elongation (hsa 00062)	0.000393862316908	5	2
9.	Adherens junction (hsa 04520)	0.000600606807405	24	3
10.	Protein processing in endoplasmic reticulum (hsa 04141)	0.000719067931089	52	4
11.	Hepatitis B (hsa 05161)	0.000831189493265	41	4
12.	Colorectal cancer (hsa 05210)	0.00105284269565	21	4
13.	Chronic myeloid leukemia (hsa 05220)	0.0011487759517	26	2
14.	Glioma (hsa 05214)	0.00117316207454	22	2
15.	Valine, leucine and isoleucine degradation (hsa 00280)	0.0014848528083	15	2
16.	Proteoglycans in cancer (hsa 05205)	0.0014848528083	53	4
17.	p53 signaling pathway (hsa 04115)	0.00271217599064	25	3
18.	Progesterone-mediated oocyte maturation (hsa 04914)	0.00299144080019	30	4
19.	Pancreatic cancer (hsa 05212)	0.00339287122318	23	2
20.	Endocytosis (hsa 04144)	0.00740160464535	55	4
21.	Valine, leucine and isoleucine biosynthesis (hsa 00290)	0.00936978210372	2	2
22.	Oocyte meiosis (hsa 04114)	0.00936978210372	31	3
23.	Sphingolipid metabolism (hsa 00600)	0.0158257005999	13	2
24.	Melanoma (hsa 05218)	0.0231238970517	20	2
25.	TNF signaling pathway (hsa 04668)	0.0273433465936	30	3
26.	Bladder cancer (hsa 05219)	0.035250285817	15	2
27.	FoxO signaling pathway (hsa 04068)	0.0377717190064	38	4
28.	Endometrial cancer (hsa 05213)	0.0377780248664	16	3

# Columns 4 and 5 of the table show the number of genes and miRNAs that have been changed

**Fig. 2:**
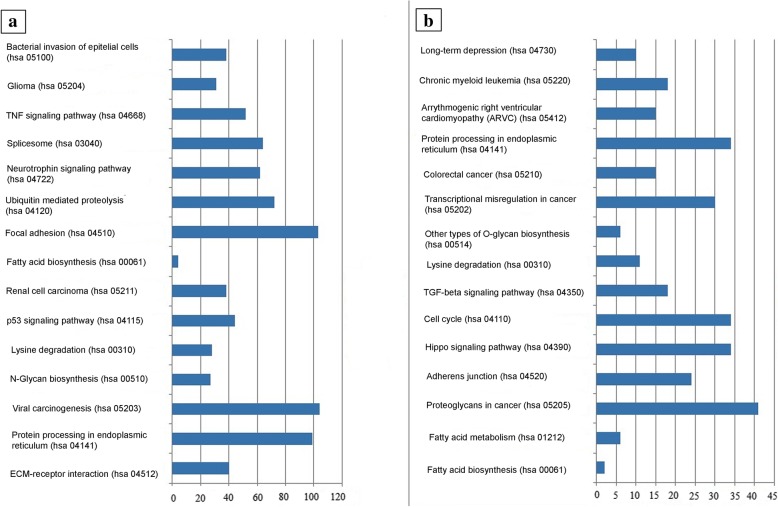
Top 15 signaling pathways targeted by (**a**) upregulated microRNAs and (**b**) downregulated microRNAs in melanoma versus healthy adjacent skin

Figure 2 shows the top 30 most significant signaling pathways for differentially expressed microRNAs for a group of melanoma and healthy adjacent tissues.

Notably, the number of dysregulated pathways in melanoma controlled by upregulated miRs was higher compared with those controlled by downregulated miRs. Moreover, there are signaling pathways controlled by downregulated microRNAs that are usually not considered in regards to cancer, such as those implicated in long-term depression and arrythmogenic right ventricular cardiomyopathy.

### qPCR

**Fig. 3:**
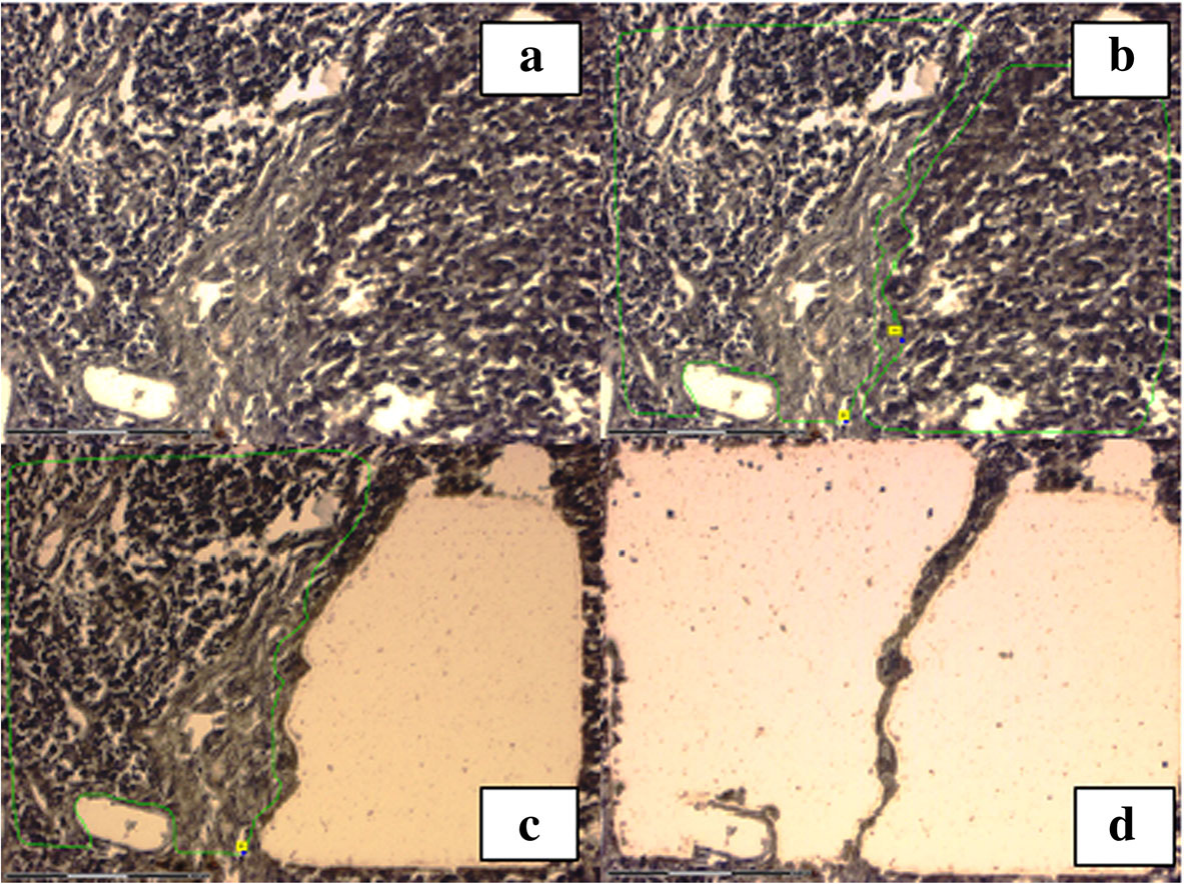
Sequential laser microdissection of melanoma tumor cells from a patient with a cutaneous primary melanoma. (**a**) Melanoma prior tomicrodissection; (**b**) the region of interest is marked with a green line; (**c** and **d**) the sample after microdissection of tumor nodes

For validation of the microarray results, microRNA sexhibiting differences inexpression between the tumor and adjacent non-canceroustissuesaccording to the microarray data (miR-363-3p, fold change 5.43; *P* = 0.023) and microRNAs with expression levels equal among the mentioned groups (miR-3591; *P* > 0.05) were selected. qPCRshowed miR-363-3p (*P* = 0.01) to be upregulated in tumor tissues. The miR-3591 expression levels were similar between melanoma and non-melanoma tissues as indicated by microarray, and qPCR produced the same results. Then, several microRNAs that exhibited differences in expression levels between melanoma cells and adjacent non-cancerous tissues were selected. The mentioned microRNAs were selectedbecause their expression rate was significantly increased in melanoma, and their target genes were related to carcinogenesis. Thus, miR-363-3p, miR-18a-5p and miR-146a-5p were studied in tissues separated by laser microdissection. Histological specimens were photographed after dissection to ensure the morphological homogeneity of the resulting material (Fig. 3).

**Fig. 4:**
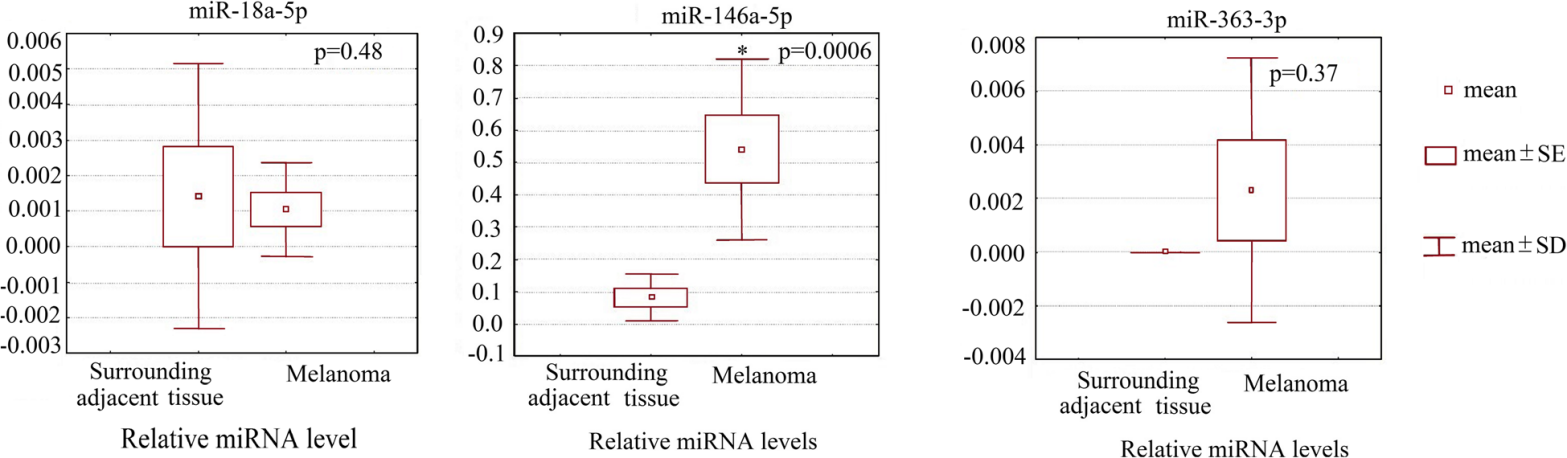
miR-18a-5p, miR-146a-5p and miR-363-3p expression levels in melanoma cells and healthy adjacent skin as evaluated by qPCR

qPCR did not reveal significant expression differences in miR-18a-5p (*P* = 0.48) and miR-363-3p (*P* = 0.37) between melanoma and non-melanoma tissues, whereas the miR-146a-5p levels were lower in adjacent non-melanoma tissues compared with melanoma (*P* = 0.0006) (Fig. 4) (Additional file 4).

### Bioinformatics analysis of miR-146a-5p for target gene and associated pathway identification

To reveal the functional role of miR-146a-5p in melanoma cells, bioinformatics analysis was carried out using the databases TargetScan 7.0, miRWalk 2.0, miRTarBase v.4.5 and miRDB v.4.0, which allowed identification of the potential target genes of this miR. A total of 36 target genes were identified for miR-146a-5p, in accordance with the data-matching programs mentioned above (Additional file 5).

The predicted targets included: NRAS (oncogene involved in cell proliferation, differentiation and adhesion regulation); IRAK1 (the gene involved in the signaling pathway of interleukin-1 and apoptosis regulation); TRAF6 (TRAF proteins mediate signal transduction from members of the TNF receptor superfamily, interact with the transforming growth factor β receptor complex, and are required for Smad-independent activation of the JNK and p38 kinases, and for NF-κB and MAPK activation); LFNG (participates in the epithelial-to-mesenchymal transition, acting as a tumor suppressor in KRAS-associated tumors);and RARB (retinoic acid receptor activator). DIANA-mirPath v.3.0 was applied to explore the signaling pathways associated with miR-146a-5p expression alterations. The main signaling pathways associated with miR-146a-5p were the Toll-like receptor signaling pathway, NF-κB signaling pathway, ErbB signaling pathway and measles signaling pathway (Table 2).

**Table 2:** Signaling pathways associated with hsa-miR-146a-5p melanoma compared with healthy adjacent skin

1 KEGG signaling pathways	2 Pathway ID	3 P
TOLL-like receptors signaling pathway	hsa04620	4.21239292662e-07
NF-κB signaling pathway	hsa04064	7.99148241829e-05
Allograft rejection	hsa05330	8.42873873828e-05
Measles signaling pathway	hsa 05162	0.00203060547588
ErbB signaling pathway	hsa 04012	0.00243401817203

## Discussion

Theroleofthe tumormicroenvironmentiscrucialincancer development and progression. Recent studies demonstratedthat growth factors secreted by the primary tumor maybe translocated by exosomes and reach metastatictarget organs,wherethey integrate in the cell plasma membrane of parenchymalorgans, leading to the development of a tumor-favoringmicroenvironment [16]. Endothelins produced by melanoma microenvironment cells regulate the balance between proliferative and differentiated states of tumor cells inmetastatic sites, as cancer cells have a less prominent proliferative buta more prominent invasive ability,and their phenotype must be backshifted to a proliferative state [17]. Rearrangement of the tumor microenvironment at metastatic sites impliesa decrease in vessel density during metastasis growth [18], activation of tumor-associated macrophages, fibroblasts andlymphocytes, followed by the release oftumor growth-promoting regulators [19].

MicroRNAs of tumor cells alter the expression of various intercellular proteins thatmay affect microenvironment components, tumor angiogenesis, immune invasion and tumor-stromal interactions after being released into the intercellular space [20]. During cancer initiation and progression, the expression levels of multiple miRNAs are aberrantly up- or downregulated, and create an imbalance in the functioning of cellular pathways [21].

MicroRNA analysis identified32 microRNAs as upregulated and 111 as downregulated in melanoma versus healthy adjacentskin. The fact that thetumor microenvironment consists of various cell types characterized by heterogeneous origin may explain its more divergent microRNA profile compared with melanoma cells.

MicroRNA profiling provided by microarray with a subsequent qPCR confirmation identified miR-146a-5p as one of the most upregulated miRs in melanoma as compared withadjacent non-melanoma tissues. An identical tendency was reported for thyroid cancer [22]. miR-146a-5p acts as an oncosuppressor in non-small-cell lung cancer, inhibiting tumor cell proliferation by direct targeting of cyclin D1 and cyclin D2 [23]. In prostate cancer cells, miR-146a-5p negatively regulates protein Rac1, which is related to cancer cell migration, thus affecting the development of metastasis [24]. By triggering signaling pathways, miR-146a-5p is implicatedindiverse processes that occur during tumor development. Recent studies determined that miR-146a-5p regulates the activity of the MAPK signaling pathway, which is one of the most important cascades in melanoma development and progression [25]. In addition, miR-146a-5p was found to beone of four microRNAs differentially expressed in metastatic melanoma versus primary tumor [26].

Our study revealed Toll-like receptor and NF-κB signaling pathway dysregulation in melanoma as compared to healthy adjacent skin. Several microRNAs were previously found to be released by tumor cells and interact with Toll-like receptors of immune cells, leading to their activation, accompanied by upregulation of NF-κB and secretion of the proinflammatory, prometastatic cytokines TNF-α and IL-6 [27]. A similar mechanism could be proposed for immune cell activation in melanoma microenvironment.

MicroRNA profiling of periampullary adenocarcinoma and adjacent stroma revealed 43 differentially expressed microRNAsthat corresponded to signaling pathways implicated inepithelial-to-mesenchymal transition, MAPK signaling andfocal adhesion [28]. miR-1 was found to be diminished in glioblastoma cells as compared to adjacent brain tissue, andit is involved in establishing interactions between glioblastoma cells, controlling angiogenesis and invasion [29]. Several investigations were conducted to evaluate microRNA profiling differences between isolated melanoma cells and the microenvironment in vitro to study their interactions [30, 31], but not in clinical samples, as in the present study.

The goal of this study was to determine microRNA expression pattern differences between melanoma cells and healthy adjacent skin, and the findings demonstrated that microRNAs involved in the basic cancer-related cellular processeswere differentially expressed between the two. When establishing significant differences between the tumor and surrounding tissues,we must take into account when high-throughput methods are applied for tumor cell characterization, as microRNAs tightly regulate mRNA expression. miR-146-5p, as other significantly altered microRNAs, requires further investigation as a possible endogenous control for accurate separation of the tumor from its adjacent tissues.

## Conclusions

Understanding the role of miR-146-a-5p in complex interactions between the tumor and the cells of its microenvironment, and in oncogenesis-related gene targeting, is necessary for elucidating the mechanisms that underlie tumor development and progression. Despite tumor cell heterogeneity and their ability to affect the gene expression pattern of the surrounding cells, making it more similar to that oftumor cells, the microRNA expression profile differs significantly between tumor and adjacent tissues.

## Additional files

Additional file 1: Differentially expressed microRNA between melanoma, healthy adjacent skin and normal skin. (XLS 127 kb)

Additional file 2: Differentially expressed microRNA in melanoma tissues compared to healthy adjacent skin according to a microarray analysis. (XLS 42 kb)

Additional file 3: Signaling pathways of the altered cluster, based on the results of the study of the expression profiles of microRNAs in melanoma, healthy adjacent skin. (DOC 56 kb)

Additional file 4: Expression levels of microRNAs in melanoma cells and healthy adjacent skin to a real-time PCR analysis. The data correspond to the graphs in Fig. 4. (DOC 29 kb)

Additional file 5: 36 target genes as identified for miR-146a-5p. (DOC 47 kb)

## Abbreviations

ANOVA: Analysis of variance
FDR: False discovery rate
MAPK: Mitogen activated protein kinase
NF-κB: nuclear factor-kappa B
PCR: Polymerase chain reaction
TGF: Transforming growth factor
TNF: Tumor necrosis factor
