# Supplementary material for: Differences in microRNA expression between melanoma and healthy adjacent skin
Source: BMC Dermatol. 2019 Jan 5;19:1. doi: 10.1186/s12895-018-0081-1 (PMC6321655; doi:10.1186/s12895-018-0081-1)
Supplement: Supplementary file 3 — Signaling pathways of the altered cluster, based on the results of the study of the expression profiles of microRNAs in melanoma, healthy adjacent skin. (DOC 56 kb) [file 12895_2018_81_MOESM3_ESM.doc]

Additional file 3. Signaling pathways of the altered cluster, based on the results of the study of the expression profiles of microRNAs in melanoma, healthy adjacent skin

| **№** | **Signaling pathway** |
| --- | --- |
| 1 | Adherens junction |
| 2 | Allograft rejection |
| 3 | Arrhythmogenic right ventricular cardiomyopathy (ARVC) |
| 4 | Bacterial invasion of epithelial cells |
| 5 | Cell cycle |
| 6 | Chronic myeloid leukemia |
| 7 | Colorectal cancer |
| 8 | ECM-receptor interaction |
| 9 | ErbB signaling pathway |
| 10 | Fatty acid biosynthesis |
| 11 | Fatty acid degradation |
| 12 | Fatty acid metabolism |
| 13 | Focal adhesion |
| 14 | FoxO signaling pathway |
| 15 | Glioma |
| 16 | Hippo signaling pathway |
| 17 | Leishmaniasis |
| 18 | Long-term depression |
| 19 | Lysine degradation |
| 20 | Melanoma |
| 21 | Measles |
| 22 | Neurotrophin signaling pathway |
| 23 | N-Glycan biosynthesis |
| 24 | Non-small cell lung cancer |
| 25 | NF-kappa B signaling pathway |
| 26 | One carbon pool by folate |
| 27 | Other types of O-glycan biosynthesis |
| 28 | p53 signaling pathway |
| 29 | Pancreatic cancer |
| 30 | Protein processing in endoplasmic reticulum |
| 31 | Proteoglycans in cancer |
| 32 | Regulation of actin cytoskeleton |
| 33 | Renall cell carcinoma |
| 34 | Ribosome |
| 35 | RNA degradation |
| 36 | RNA transport |
| 37 | Spliceosome |
| 38 | TGF-beta signaling pathway |
| 39 | TNF-signaling pathway |
| 40 | Toll-like receptor signaling pathway |
| 41 | Transcriptional misregulation in cancer |
| 42 | Ubiquitin mediated proteolysis |
| 43 | Viral carcinogenesis |
