# Supplementary material for: Differences in microRNA expression between melanoma and healthy adjacent skin
Source: BMC Dermatol. 2019 Jan 5;19:1. doi: 10.1186/s12895-018-0081-1 (PMC6321655; doi:10.1186/s12895-018-0081-1)
Supplement: Supplementary file 4 — Expression levels of microRNAs in melanoma cells and healthy adjacent skin to a real-time PCR analysis. The data correspond to the graphs in Fig. 4. (DOC 29 kb) [file 12895_2018_81_MOESM4_ESM.doc]

Additional file 4. Expression levels of microRNAs in melanoma cells and healthy adjacent skin to a real-time PCR analysis. The data correspond to the graphs in Fig. 4.

| microRNA | Relative Quantity in melanoma cells,  median [25%; 75%] | Relative Quantity in healthy noncancerous tissue,  median [25%; 75%] | P |
| --- | --- | --- | --- |
| miR-18a-5p | 0,001042 [0.00; 0.002515] | 0,001410 [0.00; 0.00000] | 0.48 |
| miR-146a-5p | 0,567081 [0.266410; 0.778188] | 0,067896 [0,020383; 0,164384] | 0.006 |
| miR-363-3p | 0.000000 [0.000000; 0.003069] | 0.000000 [0.000000; 0.000000] | 0.37 |
