# Supplementary material for: Differences in microRNA expression between melanoma and healthy adjacent skin
Source: BMC Dermatol. 2019 Jan 5;19:1. doi: 10.1186/s12895-018-0081-1 (PMC6321655; doi:10.1186/s12895-018-0081-1)
Supplement: Supplementary file 5 — 36 target genes as identified for miR-146a-5p. (DOC 47 kb) [file 12895_2018_81_MOESM5_ESM.doc]

Additional file 5. 36 target genes as identified for miR-146a-5p

| **№** | **Target genes** |
| --- | --- |
| 1 | ABL2 |
| 2 | ADAM19 |
| 3 | BCORL1 |
| 4 | C16orf52 |
| 5 | DCAF12 |
| 6 | ERBB4 |
| 7 | ERLEC1 |
| 8 | GDNF |
| 9 | HNRNPD |
| 10 | IRAK1 |
| 11 | JAZF1 |
| 12 | KDM2B |
| 13 | KIF24 |
| 14 | LFNG |
| 15 | LTB |
| 16 | MARK1 |
| 17 | MMP16 |
| 18 | NOVA1 |
| 19 | NRAS |
| 20 | NUMB |
| 21 | PRKAA2 |
| 22 | RFX7 |
| 23 | RUNX1T1 |
| 24 | SEC23IP |
| 25 | SIAH2 |
| 26 | SLCO3A1 |
| 27 | SMAD4 |
| 28 | STRBP |
| 29 | SYT1 |
| 30 | TMEM136 |
| 31 | TRAF6, |
| 32 | USP47 |
| 33 | WASF2 |
| 34 | WWC2 |
| 35 | ZBTB2 |
| 36 | ZNF512B |
